# Applicability of papain solutions in immunohematology

**DOI:** 10.31744/einstein_journal/2019AO4328

**Published:** 2019-04-22

**Authors:** Laís Priscila De Santis, Patrícia Carvalho Garcia, Valéria Nogueira Dias Paes Secco, Rosana Rossi Ferreira, Elenice Deffune

**Affiliations:** 1 Universidade Estadual Paulista “Júlio de Mesquita Filho”, Botucatu, SP, Brazil; 2 Universidade Estadual Paulista “Júlio de Mesquita Filho”, Bauru, SP, Brazil

**Keywords:** Antigens, Allergy and immunology, Papain, Enzymes, Antígenos, Alergia e imunologia, Papaína, Enzimas

## Abstract

**Objective:**

To compare the enzyme activity of different presentations of papain solution to validate in-house preparations.

**Methods:**

Two papain solutions were prepared, and the third presentation was a commercial solution. Tests were carried out with samples of red cells typed as weak RhD.

**Results:**

In-house prepared papain solutions showed similar enzyme reactivity, and statistically no differences compared to the enzyme activity of the commercial solution.

**Conclusion:**

Evaluating the cost-benefit ratio, the in-house prepared papain solutions present more economic advantages, and can be incorporated into immunohematological routines as a way to cope with periods of financial crisis and cost-containment policies.

## RESUMO

**Objetivo:**

Comparar a atividade enzimática de diferentes apresentações de solução de papaína para validação de preparados *in-house*.

**Métodos:**

Foram preparadas duas soluções de papaína, e a terceira apresentação tratou-se de uma solução comercial. Os testes comparativos das reações enzimáticas foram realizados com amostras de hemácias tipadas como RhD fraco.

**Resultados:**

As soluções de papaína preparadas *in-house* apresentaram reatividade enzimática semelhante e estatisticamente sem diferenças em comparação com a atividade enzimática da solução comercial.

**Conclusão:**

Avaliando-se a relação entre custo e benefício, as soluções de papaína preparadas *in-house* são economicamente vantajosas, podendo ser incorporadas às rotinas imuno-hematológicas como forma de enfrentamento em períodos de crise financeira e em políticas de retenção de gastos.

## INTRODUCTION

The red blood cell membrane is composed of a variety of proteins that anchor themselves or transverse the lipid bilayer. Many are polymorphic, and define several types of blood groups by differentiating antigens linked to them.^(^ ^1^ ^–^ ^3^ ^)^ According to the International Society of Blood Transfusion (ISBT), the antigens that characterize the blood groups can be found in one of the four classifications. Currently, there are 36 blood group systems, 38 antigens that have not yet been attributed to any system, 15 antigens distributed among 6 collections, 17 low-frequency antigens, and 6 high-frequency antigens.^(^ ^4^ ^–^ ^8^ ^)^

Considering the significant genetic polymorphism of the blood groups, the heterogeneity of presentations of antigens on membranes of red blood cells, and the complexity of each case to identify irregular antibodies of blood donor and recipients, immunohematology draws on laboratory supplies that recognize specific membrane structures, or techniques that increase agglutination of red blood cells, or modify/eliminate the expression of antigens or protein structures of the red blood cells.^(^ ^9^ ^)^ The combination of these techniques helps in the correct identification of antibodies in routine immunohematology tests, or in the identification of antigens – be them rare or not - on the membrane of donor and recipient red blood cells. Among these techniques, one can list the use of monoclonal antibodies, reagents such as dithiothreitol (DTT), chloroquine diphosphate, low ionic strength solutions (LISS), and proteolytic enzymes.^(^ ^9^ ^)^

### Action of the proteolytic enzymes on the membrane of red blood cells

The activity of each reagent or proteolytic enzyme is different on each antigen (Table 1). Taking into account the expression of a blood group antigen is determined by its biochemical structure and by the position relative to the lipid bilayer, in spite of a previous enzyme treatment of red blood cells, *e.g.,* some sensitive antigens may be destroyed or become weakly reactive, or, yet, begin having a more intense interaction with specific antibodies.^(^ ^10^ ^)^

**Table 1:** Effects of enzymes and chemical solutions on some of the main antigens and proteins of the red blood cell membrane

Antigens/proteins	Enzymes/chemical solutions					
	Papain	Bromelain	Trypsin	Alpha-chymotrypsin	Ficin	DTT
A	+	+	+	+	+	0
B	+	+	+	+	+	0
H	+	+	+	+	+	0
D	+	+	+	+	+	0
C	+	+	+	+	+	0
C	+	+	+	+	+	0
E	+	+	+	+	+	0
E	+	+	+	+	+	0
K	0	0	0*	0*	0	-
K	0	0	0*	0*	0	-
Fy^a^	-	-	0	-	-	0
Fy^b^	-	-	0	-	-	0
Fy^6^	-	-	0	-	-	0
Fy^3^	-	-	0	-	-	0
Jk^a^	+	+	+	+	+	0
Jk^b^	+	+	+	+	+	0
Jk^3^	+	+	+	+	+	0
Le^a^	+	+	+	+	+	0
Le^b^	+	+	+	+	+	0
M	-	-	-	0	-	0
N	-	-	0	-/0	-	0
S	-/0	-	0	-	-/0	0
S	-/0	-	0	-	-/0	0
Di^a^	0	+	0	0	0	0
Di^b^	0	+	0	0	0	0
P	+	+	+		+	0
I	+	+	+	+	+	0
Lu^a^	0	0	-	-	0	W/-
Lu^b^	0	0	-	-	0	W/-
Do^a^	+	+	-	W	+	-
Do^b^	+	+	-	W	+	-
Vel	0	0	0	0	0	0/-

Source: Antibody detection, identification, and compatibility testing — introduction. Method 3-11. Evaluating enzyme-treated red cells. In: Fung MK, Grossman BJ, Hillyer CD, Westhoff CM, editors. American Association of Blood Banks (AABB). Technical Manual. 19th ed. EUA: AABB; 2017;^(^^11^^)^ Girello AL, Kuhn TI. Pesquisa e identificação de anticorpos irregulares. In: Girello AL, Kuhn TI. Fundamentos da Imuno-hematologia eritrocitária. 3a ed. São Paulo: Senac; 2011. p.101-26;^(^^12^^)^ Castilho L, Pellegrino Júnior J, Reid ME. Fundamentos de imuno-hematologia. São Paulo: Atheneu; 2015. Sistemas de grupos sanguíneos com base em proteínas de passagem única. p. 45-15.^(^^13^^)^

* Antigens of the Kell system are sensitive to treatment with mixture of trypsin and alpha-chymotrypsin. + antigens with intensified reactivity; 0: antigens that suffer no interference; - sensitive antigens, that can be destroyed; -/0 some antigens are destroyed or suffer no interference.

DTT: dithiothreitol; W: weak antigens.

The carboxylic groups of the sialoglycoproteins comprising part of red blood cell membranes are the primary factor responsible for the negative charge on the surface of the red blood cell; and when in a solution, they create an electric potential (zeta potential).^(^ ^14^ ^)^ Enzyme treatment with papain (that comes from papaya), bromelain (from the pineapple), trypsin, alpha-chymotrypsin (both of animal origin), or ficin (from figs), in addition to other techniques, modifies the electronegativity, and allows the red blood cells to become agglutinable.^(^ ^13^ ^,^ ^14^ ^)^ The use of proteolytic enzymes is a useful tool for characterizing an antigen of red blood cell membrane, since it has an action of fragmenting specific peptide bonds among the amino acids that make up the proteins.^(^ ^13^ ^,^ ^14^ ^)^

There are well-defined protocols that allow the immunohematology services to prepare the in-house reagents and enzyme solutions for application in the laboratory routine. Papain, *e.g.,* an enzyme frequently used on the test bench that exerts a significant activity on a wide group of membrane antigens, may have the solution prepared at their own laboratory service, based on the consultation of protocols, such as the one presented by the Technical Manual of the American Association of Blood Banks (AABB).^(^ ^15^ ^)^ For the in-house prepared papain solutions, their use should be standardized (incubation time with the red blood cells, concentration, volume to be used, among other parameters), as well as how to validate the enzyme activity in comparison to similar commercial products. The costs of acquisition and preparation, and the time dispensed in its manipulation, should also be taken into consideration in every assessment.

As a consequence of the phenotypic diversity of human red blood cells, of laboratory challenges to identify erythrocyte antigens and antibodies from blood donors and patients, and of the constant need for use of laboratory supplies with more accessible values, and with an equivalent performance, this study proposed the validation of in-house preparations of papain solutions for application in the immunohematology laboratory routine.

## OBJECTIVE

To validate the in-house preparations of papain solutions for application in the immunohematology laboratory routine.

## METHODS

### Preparation of the solutions and standardization of the incubation time

**Figure 1:**
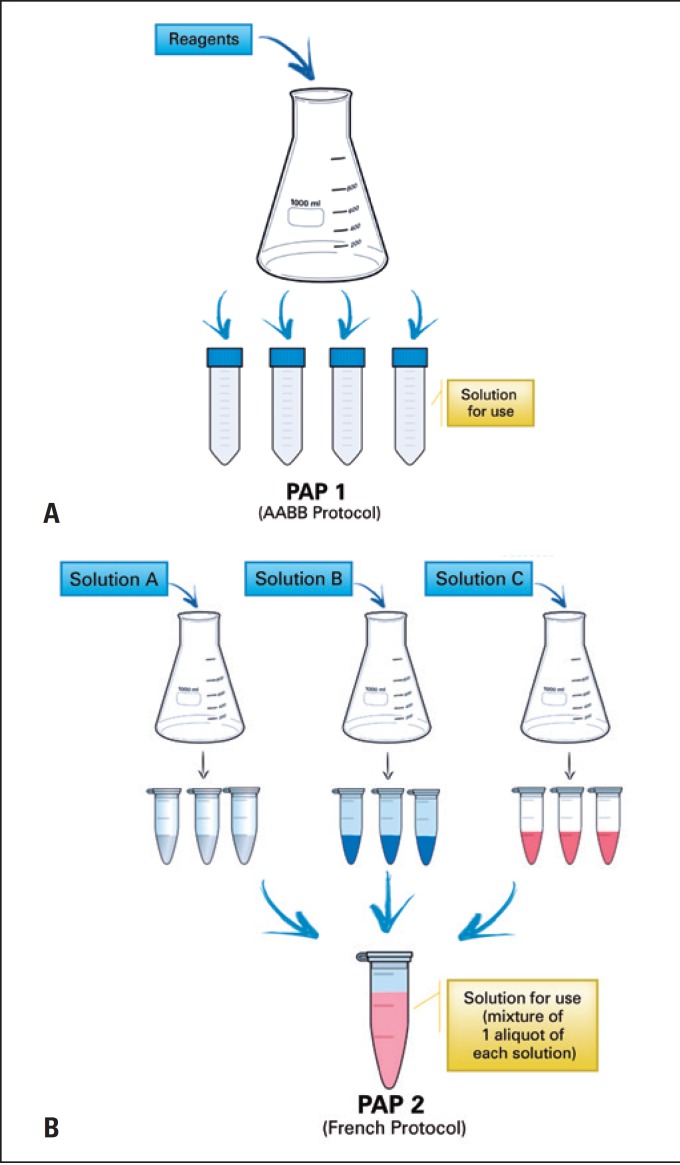
Preparation outline of the of the papain solutions (A) Preparation of PAP1, as per protocol of the American Association of Blood Banks; (B) preparation of PAP2, as per French protocols.

Source: https://encrypted-tbn0.gstatic.com/images?q=tbn:ANd9GcRSh37a69QbHlWx8rjc_P2y49-6f-Il1mCHtyUp982kkzSgSVBNjblbSyw; http://www.clker.com/clipart-closed-eppendorf-tube-white.html; http://www.clker.com/clipart-centrifuge-falcon-tube-.html#; https://images.comunidades.net/ond/ondecomeremcamacari/setabaixo.png; https://3.bp.blogspot.com/-1uGF8vCHtUU/WMp3XT8mwBI/AAAAAAAAUx8/5cqxmpc3xeQkx9-3136eLuHfP4hQmcsYQCLcB/s1600/seta_red.png

The solutions of the enzyme papain used were a commercial product, ID-Papain, Bio-Rad™ (PAP3) and two prepared in-house based on two distinct protocols. The 1% papain solution (PAP1) was prepared as per description in the Technical Manual of AABB.^(^ ^15^ ^)^ PAP1 had the enzyme activated at the time of its preparation and was maintained frozen, and its use could be made after defrosting. The method of preparation of the 0.125% papain solution (PAP2) was based on French protocols. Three distinct solutions and activation of the enzyme occur at the time of its use, after defrosting and mixture of one aliquot of each solution. Figure 1 presents a detailed illustration of preparation of both papain solutions.

For the standardization of incubation time of the red blood cells with papain, a sample of weak RhD-positive red blood cells, obtained at the Donor Immunohematology Unit, of *Hemocentro do Hospital das Clínicas da Faculdade de Medicina de Botucatu*. The choice of this phenotype was made because the agglutination reactions involving antigens of the Rh blood group were intensified after treatment of the red blood cells with papain. The blood sample was washed three times in a 0.9% isotonic saline solution (ISS); a 5% suspension in ISS was prepared; in a test tube, a previous test was performed to verify the intensity of agglutination with antibody anti-D from the non-treated sample. The analysis of all agglutination reactions carried out in this study was visual, observing the consistency of the lumps formed by agglutination of red blood cells, as per the criterion determined by Race et al.,^(^ ^16^ ^)^ and modified by Marsh.^(^ ^17^ ^)^

The standardization of incubation time was done, volume by volume, of PAP1 and PAP2 with the concentrate of washed weak RhD red blood cells, followed by incubation in a water bath at 37°C. Each tube of each solution of papain+red blood cells was incubated at three different times (3, 5, 7, and 10 minutes). After incubation, the solution was washed three times with ISS, and tests with anti-D were performed. For comparative tests, a sample of red blood cells in a 5% solution in ISS was prepared, and then proceeded to treatment with a commercial papain, according to the manufacturer’s instructions. After treatment of red blood cells, the reaction was verified using anti-D monoclonal antibody.

### Comparative tests of the enzyme activity of papain solutions

In order to verify if the papain solutions had the same enzyme activity on the red blood cell membranes, tests between the papain solutions were performed using samples of weak RhD red blood cells (n=6). The cells were washed three times with ISS, having gone through incubations of red blood cells with PAP1 and PAP2, as per the standardization parameters, and PAP3, following the manufacturer’s instructions, plus a posterior test with anti-D antibody on treated and untreated red blood cells. Based on the classification of agglutination intensities, statistical analysis was carried out using Student’s *t* test to evaluate if the enzyme activity of each preparation of papain has a statistically significant difference (p≤0.05) and can have its activities equated.

The article was approved by the Research Ethics Committee, opinion no. 1.622.156, CAAE: 56511116. 5.0000.5411.

## RESULTS

In the reactions observed in incubation time standardization, the intensity of agglutination of weak RhD red blood cells that did not undergo enzyme treatment was smaller than that of red blood cells previously treated with an enzyme (Table 2). In a descending analysis to define the better treatment in this test, analyzing the solidity and intensity of agglutinations, one can establish that PAP3 > PAP2 > PAP1.

**Table 2:** Performance assessment of three different presentations of papain for use in immunohematology in incubation time standardization tests

Type of treatment	Intensity of reaction					Score				
		Time								
		3’	5’	7’	10’		3’	5’	7’	10’
Without treatment	1+/2+					5/8				
PAP1		3+	3+*	2+/3+	2+/3+		10	10	8/10	8/10
PAP2		3+	3+	3+/4+*	2+/3+		10	10	10/12	8/10
PAP3			4+					12		

Intensity of agglutination classified as crosses and score.

* Most solid button compared to others (better time for sample). PAP1: American Association of Blood Banks protocol; PAP2: French protocol (three aliquots with activation of enzyme at the time of use); PAP3: commercial Bio-Rad™.

The ideal incubation time between the papain solution and red blood cells, at a temperature of 37°C, for treatment of red blood cells with PAP1 was 5 minutes, while for PAP2, the ideal time was 7 minutes.

**Figure 2:**
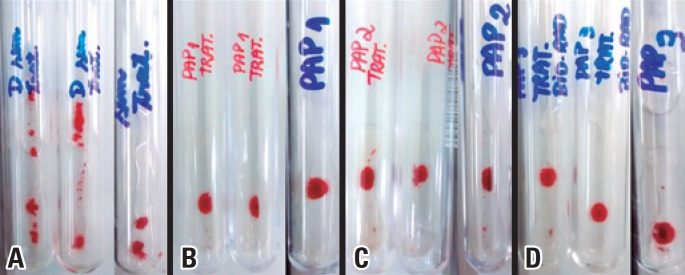
Some agglutination reactions observed with weak RhD red blood cells, both untreated and treated with different solutions of papain. (A) reactivity of weak RhD samples without treatment; (B) reactivity of weak RhD samples treated with PAP1; (C) reactivity of weak RhD samples treated with PAP2; (D) reactivity of weak RhD samples treated with PAP3

The treatments given in six weak RhD red blood cell samples, with the three presentations of papain solution, were analyzed to assess equivalence of enzyme activity among PAP1, PAP2, and PAP3. They resulted in reactions considered equivalent, as shown in figure 2.

The statistical analysis of the results using Student’s *t* test showed that there is a statistically significant difference between the samples that did not undergo enzymatic treatment, and those treated with any one of the presentations of papain (Table 3). Nevertheless, there were no statistical differences between the in-house produced papain (PAP1 and PAP2) and the commercial papain, which demonstrates that, considering antigen D of red blood cell membrane, the enzyme activity of any papain presentations has the same performance.

**Table 3:** Statistical analysis as per the Student’s *t* test for enzyme treatments

Parameters	No treatment *versus* PAP1	No treatment *versus* PAP2	No treatment *versus* PAP3	PAP1 *versus* PAP2	PAP1 *versus* PAP3	PAP2 *versus* PAP3
p value	0.0009	0.0027	0.0121	0.3632	0.2353	0.2480

Table 4 displays information about each papain preparation. The investigation of preparation and acquisition costs took into consideration the values of the reagents and hours of work of a biomedical professional. An agglutination column test (gel test) was performed, in which 1% suspensions of red blood cells and a proportional volume of papain solution of papain would be required for this quantity of red blood cells to treat them with the enzyme.

**Table 4:** Characteristics of each presentation of papain solution

Characteristics	Type of presentation		
Papain	PAP1	PAP2	PAP3
Presentation	Solution prepared from freeze-dried enzyme. Ready for use after thawing	Preparation of buffer solutions and of aliquots I, II and III. For use, each aliquot is mixed after thawing	Liquid – ready for use
Storage	-80°C	-80°C	+4°C
Preparation time	2 hours	1 hour	-
Validity	5 years	5 years	7 weeks
Equipment required for preparation	Centrifuge, agitator, water bath, freezer -80°C	Agitator, freezer -80°C	-
Costs	200mL=R$ 126,00 10mL=R$ 6,30	400mL=R$ 355,47 10mL=R$ 8,89	10mL=R$ 81,40*

* *Hemocentro de Botucatu* procurement data - search in January 2017.

## DISCUSSION

Each preparation protocol presented with distinct characteristics that can be interpreted as advantages and/or disadvantages in each method.

Relative to durability, PAP3 solution has a sort shelf life. The datasheet of the product informs that the validity, as of the manufacturing date, is 7 weeks, since it is presented in the liquid form. This fact may be considered limiting for the use of this reagent, because it requires a programmed purchase and monthly delivery. Whereas the PAP1 and PAP2 solutions, stored at -80°C, maintain efficient reactivity for up to 5 years.

Current laws allow blood banks to produce reagents for use on the test bench. According to Article 21 of the Consolidation Ordinance No. 5, dated 28 September 2017, Annex IV, “The blood bank is allowed to produce and use reagents for immunohematological tests, as long as there is authorization by ANVISA [Brazilian Health Regulatory Agency], as provided in Article 6, Law no. 10,205, from 2001”.^(^ ^18^ ^)^

Law No. 10,205^(^ ^19^ ^)^ regulates the 4^th^ paragraph of Article 199 of the Federal Constitution, and sets forth in its sixth article that:

All materials and substances or related products that have direct contact with blood collected for transfusion purposes, as well as reagents and supplies for laboratory, and employed to comply with the Technical Rules, should be registered or authorized by the Health Surveillance Agency of the Ministry of Health (Presidency of the Republic, 2001).

The analysis of costs in the preparation of PAP1 and PAP2 solutions reveals implementing these solutions in the laboratory routine would be viable, since 10mL of PAP3 is very costly for acquisition, compared with the costs for 10mL of PAP1 or PAP2 solutions. However, one should take into consideration the time necessary to prepare the enzyme for use on the test bench, since the laboratory professionals should dedicate to this activity, besides requiring, on the part of the department, authorization from the Health Surveillance agencies, with the same objective, *i.e.*, maximum quality of the reagents, supplies, and services rendered.

## CONCLUSION

The existence of protocols for in-house preparation of supplies enables a decrease in expenses with health services, especially those that participate in the area of immunohematology, and guarantee the same quality as those commercially available, with the backing of regulatory goals. The preparation time of the papain solutions was, on average, 2 hours of work for the production of up to 400mL of solution, which had a shelf life of 5 years. Additionally, based on the test performed, with reaction to antigen D, one can affirm that homemade papain has the same enzyme efficiency, and can be adopted in the laboratory routine with safety and quality assurance.

The decrease in costs can also be reached with the in-house mounting of phenotyped red blood cell panels for research, and identification of irregular antibodies, routine tests of immunohematology laboratories and blood banks.

Therefore, the decrease in costs of the laboratory tests depends on the initiative of each service in validating the possible exchange with statistical methods, and the evaluation of costs and benefits of exchanging the commercial product for an in-house prepared item. The values saved could be applied in the acquisition of new equipment, implementing new assays, technical training, and primarily, in reverting this improvement to the population that depends on public healthcare services.
